# Can the different presentation of the diabetic ischaemic foot be explained by different distributions of arterial disease

**DOI:** 10.1186/1757-1146-3-S1-O19

**Published:** 2010-12-20

**Authors:** Elizabeth Pendry, Hisham Rashid, Michael Edmonds, Rosalind Phelan

**Affiliations:** 1 Kings College Hospital, London, UK

## Background and aims

It is difficult to understand the various presentations of the diabetic ischaemic foot. The aim of the study was to investigate the clinical and angiological features of three presentations of the diabetic ischaemic foot: the painful ischaemic foot with tissue loss (group 1), the painful ischaemic foot with no tissue loss (group 2), and the painless ischaemic foot with tissue loss (group 3). The hypothesis was that Group 1 patients would have more extensive distribution.

## Methods

We studied 10 patients who presented with unilateral forefoot ischaemic rest pain and unilateral tissue loss (Group 1), 9 patients who presented with unilateral forefoot rest pain but no tissue loss (Group 2) and 10 patients who presented with unilateral tissue loss but no rest pain (Group 3). Ischaemia was determined by absent foot pulses and damped Doppler waveforms of foot arteries. All patients underwent contrast angiography of lower limbs. The angiograms were examined for >50% stenosis or occlusion in the following arterial levels: iliac, common femoral (CFA), superficial femoral (SFA), profunda, popliteal and tibial. For each patient, the individual levels of arterial disease and the number of separate arterial levels were assessed.

## Results

The mean age was similar in the 3 groups: in group 1, mean age was 69.5±14.5 years (mean ±SD), in group 2-mean age was 72.4±10.6 years and in group 3-mean age was 70.8±11.3 years, p=0.879. There was no significant difference in the distribution of the arterial levels in each group: in group 1 the distribution was iliac 1/10, SFA 5/10, popliteal 4/10 and tibial 8/10; in group 2 – iliac 2/9, CFA 1/9, SFA 8/9, profunda 1/9, popliteal 2/9 and tibial 6/9; in group 3 – iliac 2/10, SFA 7/10, popliteal 4/10 and tibial 9/10. There was no significant difference in the number of arterial levels in each group. In Group 1, there was 1.7±0.5 arterial levels, in Group 2 - 2.2±0.8 arterial levels and in group 3 also 2.2±0.7 arterial levels, p=0.159. The mean serum creatinine was similar in the 3 groups: Group 1-125.7±42.4µmol/L, group 2 -149.0 ±102.2µmol/L and group 3 – 120.6 µmol/L± 79.8, p=0.705.

## Conclusion

There is a similar distribution of arterial disease in patients presenting with a painful ischaemic foot and tissue loss, painful ischaemic foot with no tissue loss, and painless ischaemic foot with tissue loss. Thus these clinical presentations are not determined determined by the extent of arterial disease.

